# Left digit bias in children’s and adults’ paper-and-pencil number line estimation

**DOI:** 10.3758/s13421-025-01707-y

**Published:** 2025-04-09

**Authors:** Hilary Barth, Bethany Rutkowski, Leah Vaidya, Erin Kim, Cameron Bourassa, Annie Fabian, Sierra Eisen, Alexandra Zax, Katherine Williams, Andrea L. Patalano

**Affiliations:** Department of Psychology, Wesleyan University, 207 High Street, Middletown, CT 06459 USA

**Keywords:** Numerical cognition, Estimation, Number line, Left digit effect, Left digit bias

## Abstract

Number line estimation tasks are frequently used to learn about numerical thinking, learning, and development. These tasks are often interpreted as though estimates are determined by overall magnitudes of target numerals, rather than specific instantiating digits. Yet estimates are strongly biased by leftmost digits. For example, numbers like “698” are placed too far to the left of numbers like “701” on a 0–1,000 line. This “left digit effect” or “left digit bias” has been investigated little in children, and only on electronic tasks. Here, we ask whether left digit bias appears in paper-and-pencil estimates, and whether it differs for paper-based versus computer-based tasks. In Study 1, 5- to 8-year-old children completed a 0–100 number line task on paper. In Study 2, 7- to 11-year-olds completed a 0–1,000 paper task. In Study 3, adults completed tasks on paper in both ranges. Large left digit effects were observed for children aged 8 years or older and adults, but we did not find evidence for left digit bias in younger children. Study 4 compared paper and computer tasks for adults and children aged 9–12 years. Strong left digit bias was observed in all conditions, with a larger effect for the paper-based task in children. Large left digit effects in number line estimation emerge regardless of task format, with a developmental trajectory broadly consistent with other studies. For children in the age range that reliably exhibits left digit bias (but not adults), paper-and-pencil number line estimation tasks elicit even greater bias than computer-based tasks.

## Introduction

Understanding the meanings of Arabic numerals and number words is an important developmental milestone. One method frequently used to assess and guide children’s developing number understanding is the number line estimation task. In a standard number line estimation task (e.g., Barth & Paladino, 2011; Booth & Siegler, 2006; Siegler & Opfer, 2003), participants are asked to estimate the appropriate position of a numerical quantity, often represented by an Arabic numeral, on a bounded response line (e.g., with endpoints of 0 and 1,000). Children’s performance on the number line task is often used to evaluate various components of numerical knowledge (e.g., Cohen & Sarnecka, 2014; Ramani & Siegler, 2008; Rouder & Geary, 2014; Siegler, 2016; Slusser et al., 2013). Number line estimation has been shown to correlate with children’s overall mathematical competence in a wide range of studies (Schneider et al., 2018) and is predictive of arithmetic (Siegler, 2016) and precise calculation skills (Xenidou-Dervou et al., 2015). Interventions using these tasks have also led to improvements in fraction knowledge during grade school, a time when many children struggle to develop foundational fraction skills (Barbieri et al., 2020; Gunderson et al., 2019; Hamdan & Gunderson, 2017; Hansen et al., 2015).

Explorations of the malleability of number line estimation have found that seemingly unimportant task properties may affect performance patterns in meaningful ways (e.g., Dackermann et al., 2018; Zax et al., 2019). Often, number line performance has been assessed as though the numerical magnitudes of target numerals are key determinants of placements. That is, though other factors affecting placements have been widely explored too, numerals with nearly identical magnitudes were generally expected to result in similar placements. If so, the particular digits that instantiate the presented target numerals should not matter much; the numerical magnitudes of those targets would be the important factor. But recent evidence shows that placements for target numerals depend heavily on the particular digits that comprise those numerals, in the sense that numerals with nearly identical magnitudes are estimated quite differently when they have different leftmost digits. For example, when children from the ages of 7 to 11 years and adults were given a 0–1,000 number line task that included target numerals on either side of a hundreds boundary (e.g., 698 and 702), they placed them more differently than would be expected based on their very similar magnitudes (Lai et al., 2018). Both children and adults reliably placed numerals just above hundreds boundaries far to the right of numerals just below hundreds boundaries, with large effect sizes at all ages. In contrast, numerals with a similar difference in magnitude but the same left digit, such as 849 and 853, showed no analogous effects. Similarly, when tested on a 0–100 number line, children around 8 years or older and adults placed target numerals on either side of decade boundaries (e.g., 59 and 61) more differently than is warranted by their magnitudes, with the larger numeral systematically to the right of the smaller (e.g., Savelkouls et al., 2020; Williams et al., 2022; see also Patalano et al., 2023). These studies demonstrate a “left digit effect” or “left digit bias” in number line estimation. This strong left digit bias is likely to matter a good deal to the interpretation of number line task performance patterns, perhaps especially in child studies. Studies with children often use fewer trials and target numerals, so the use of particular targets might have a greater influence on findings and interpretations.

The identification of strong left digit bias in the kinds of common number line tasks employed in research and educational contexts is fairly recent (e.g., Lai et al., 2018; Savelkouls et al., 2020; Williams et al., 2020, 2022). Related forms of left digit bias are evident in various forms of decision making and have been broadly documented. For example, left digit effects appear in price comparison. Items that are priced one cent below a dollar amount (e.g., $2.99) are perceived to be a significantly lower price than those priced one cent higher (e.g., $3.00) because of the difference in the left digit, rather than a meaningful difference between the two prices (Manning & Sprott, 2009; Thomas & Morwitz, 2005). Left digit effects also appear in the context of house sales (Beracha & Seiler, 2015), smokers’ motivation to quit smoking (MacKillop et al., 2014), anticipated guilt about calories (Kim, 2020), medical professionals’ choices about eligibility for heart surgery based on age (Olenski et al., 2020), and many other areas of decision making.

Understanding left digit bias in numerical estimation, like understanding left digit bias in decision contexts, is important for practical and theoretical reasons. Number line estimation is widely used to draw conclusions about the nature and development of numerical reasoning and to understand foundational numerical and spatial skills and abilities that contribute to mathematics learning and achievement (e.g., Sutherland et al., 2021). It is therefore important to understand the task elements and other influences that shape people’s estimation performance over development. Fine-grained analyses of patterns of left digit bias in numerical estimation (Patalano et al., 2023) can also constrain models of numerical processing that are longstanding subjects of debate (Dehaene et al., 1990; Dotan & Dehaene, 2020; Huber et al., 2016a, b; see Patalano et al., 2023, for discussion).

The finding of strong left digit bias in number line estimation is, in itself, evidence that task elements that initially seem unimportant may turn out to be important. Both pencil/paper (e.g., Barth et al., 2016; Barth & Paladino, 2011; Berteletti et al., 2010; Booth & Siegler, 2006) and electronically administered number line tasks (e.g., Ashcraft & Moore, 2012; Chesney & Matthews, 2013; Hurst et al., 2014) are common, but almost no work to date has asked if paper tasks elicit left digit bias. While there is no obvious reason to predict that paper tasks should fail to elicit this bias, left digit bias on a paper-based version of the task has been demonstrated only in one study including a fairly small number of adults – and in that study, one group did and one group didn’t show the bias (Savelkouls et al., 2020). No studies have asked whether children’s pencil-and-paper number line placements are biased by leftmost digits. Yet paper-based number line tasks are widespread in research with children (e.g., Barth et al., 2016; Booth & Siegler, 2006; Dackermann et al., 2018; Honoré & Noël, 2016; Link et al., 2014; Luwel et al., 2018; Opfer & Siegler, 2007; Peeters et al., 2016; Peeters, Sekeris et al., 2017a, Peeters, Verschaffel et al., 2017b; Sasanguie et al., 2012, 2013), and there is clear evidence that seemingly small alterations in task formats and procedures can influence number line placements, both in children and in adults (e.g., Dackermann et al., 2018; Peeters et al., 2016; Peeters, Sekeris et al., 2017a).

A separate question is whether or not the degree of left digit bias is affected by number line task format. While explorations of performance on paper-based versus computer-based math tasks have been carried out in areas considerably different from the present work (e.g., Bayazit & Askar, 2012; Clariana & Wallace, 2002; Eid, 2005; Flowers et al., 2011; Johnson & Green, 2006; Keng et al., 2008; Logan, 2015; McClelland & Cuevas, 2020; Poggio et al., 2005; Tarasuik et al., 2017), there is not much published evidence using more closely related tasks. Potential influences of these formats have been explored for some elements of number line estimation performance but not others. Piatt et al. (2016) demonstrated that sixth graders’ accuracy on number line estimation tasks did not differ between paper and electronic tablet versions of the task; however, placement accuracy is distinct and dissociable from left digit bias, so left digit bias was not specifically addressed in that work. In work that specifically explored left digit effects in number line estimation, participants were tested either on a desktop or laptop computer or on a touchscreen tablet (e.g., Lai et al., 2018; Patalano et al., 2022; Williams et al., 2020, 2022, 2023), but paper tasks were not included, nor were task formats directly compared.

The primary goals of the current work are to determine whether left digit bias in number line estimation is present in children’s and adults’ performance on paper-and-pencil tasks (Studies 1–3), and whether this bias differs for paper-based versus computer-based tasks in children and adults (Study 4). A secondary goal is to better establish and explore left digit effects in children. While findings of strong adult left digit bias in number line estimation have been replicated many times in a wide range of contexts (Gwiazda et al., 2024; Kayton et al., 2022; Lai et al., 2018; Savelkouls et al., 2020; Patalano et al., 2022; Williams et al., 2020, 2022, 2023), far fewer examples are available with child participants (Lai et al., 2018; Williams et al., 2022). Accordingly, here we explore the apparent emergence and development of left digit bias in number line estimation in a novel task format (Studies 1–2; see also Study 4). The child studies described earlier reveal a developmental progression in which evidence for left digit effects in number line estimation is apparent at around age 7 to 8 years (at least for children who are exposed to mathematics learning sequences typical in the USA). These are large effects that persist into middle childhood and presumably beyond, and continue in adulthood (Lai et al., 2018; Williams et al., 2022, 2023). For a relatively large numerical range (0–1,000), children aged 7 or 8 years through 11 years and adults made estimates that were strongly influenced by the leftmost digits of three-digit numerals, with large effect sizes at all ages (Lai et al., 2018). For a relatively small numerical range (0–100), children aged 8–11 years and adults were also influenced by the leftmost digits of two-digit numerals (Williams et al., 2022). Younger children (5 and 6 years old, not tested on the larger range) exhibited no evidence of a left digit effect for two-digit numerals (Williams et al., 2022). Collectively, these findings reflect a similar developmental trajectory with respect to the apparent emergence and persistence of left digit bias. The present study aims to provide another potential example of the basic finding of left digit bias in children’s number line estimation, while asking whether a structurally similar but differently formatted task reveals a similar developmental trajectory.

We incorporated an additional minor goal, after designing the study and collecting some of the data, to follow up on a different developmental question. In addition to demonstrating a developmentally widespread left digit bias in number line estimation, previously published studies reveal cases in which numerals with very different magnitudes fail to be differentiated at all when they share a leftmost digit. For a larger numerical range (0–1,000), young children (7 or 8 years old) did not differentiate numerals with the same left digit but very different magnitudes, such as 701 and 799, whereas older children (9 to 11 years old) and adults successfully differentiated these numerals (Lai et al., 2018). The youngest children in that 0–1,000 study apparently relied *solely* on the leftmost digits for their estimates, while the older children and adults were instead strongly influenced by the leftmost digits without relying on them solely. However, for even younger children with a smaller range (0–100), no parallel pattern was observed: there was no evidence that children first relied solely on leftmost digits in this range (Williams et al., 2022). While the current studies were not designed with this purpose in mind, after observing these patterns in other data we investigated similar patterns in performance on the current paper-based tasks (Studies 1–2).

We address these questions in four studies. In Study 1, we examined whether 5- through 8-year-olds demonstrate a left digit effect on a standard paper-based number line task using the smaller 0–100 numerical range. In Study 2, we extended this question to a larger numerical range with slightly older children and examined whether 7- through 11-year-olds demonstrate a left digit effect on a standard paper 0–1,000 task. In Study 3, we investigated adults’ performance on paper versions of both the standard 0–100 and 0–1,000 tasks, within participants. In Study 4, we presented new groups of children and adults with both paper-based and computer-based number line tasks, within participants, assessing left digit bias for both task formats and asking whether the degree of bias differed between formats.

## Study 1

In Study 1, children aged 5 through 8 years completed a standard 0–100 number line task on paper. Children in this age range were presented with a 0–100 numerical range to align with previous research both on left digit effects in number line estimation (Williams et al., 2022) and on number line performance more broadly. Our primary question was whether children in each age group would display a left digit effect. For target numeral pairs on either side of decade boundaries (e.g., 69 and 71 on either side of decade boundary 70), we asked whether the larger numeral in each pair was placed farther to the right of the smaller numeral than would be expected based on their absolute differences. This study was designed in parallel with a corresponding electronically-administered 0–100 number line task that was published previously (Experiment 1, Williams at al., 2022). Though analysis plans were not formally preregistered for Studies 1–3, our main analyses were planned as reported in advance of data collection, following previous work (Lai et al., 2018).

### Methods

#### Participants

Ninety-six children were recruited from a university laboratory participant database and a local science center. Thirteen children were excluded for non-compliance (one 5-year-old, four 6-year-olds, one 7-year-old), experimenter error (two 5-year-olds, one 7-year-old), or producing estimates uncorrelated with the target numerals (two 5-year-olds, one 6-year-old). One additional 8-year-old was excluded during a later stage of data analysis for missing more than five critical pairs (see below). Our sample after these exclusions included 22 5-year-olds (*M*_*age*_ = 5;6, range = 5;2 – 5;11, 12 female, ten male), 21 6-year-olds (*M*_*age*_ = 6;7, range = 6;0 – 6;11, 12 female, nine male), 24 7-year-olds (*M*_*age*_ = 7;5, range = 7;0 – 7;11, 17 female, seven male), and 17 8-year-olds (*M*_*age*_ = 8;7 , range = 8;0 – 8;11, ten female, six male).

#### Stimuli

The number line task was completed on paper. On each trial, participants saw a horizontal line with endpoints labeled 0 (left) and 100 (right) printed on a sheet of paper in a stapled booklet. The response line was 24.5 cm long, such that a single unit in this 0–100 task was equivalent to 2.45 mm. The task was to mark the line using a pencil to indicate the appropriate location of a target numeral presented by the experimenter on a small round label. Participants completed one block of 30 trials. No feedback was provided and there were no training trials in order to avoid potential influences on placement patterns. The following target numerals were presented in a different random order to each participant: 2, 4, 8, 12, 17, 18, 22, 23, 29, 31, 36, 38, 42, 47, 49, 51, 58, 59, 61, 62, 69, 71, 74, 78, 82, 86, 88, 92, 97, 99. Eight critical between-decade two-digit target numeral pairs were embedded within this set to assess left digit effects: 18/22, 29/31, 38/42, 49/51, 59/61, 69/71, 78/82, 88/92. The target numerals in these pairs fell just above and below decade boundaries, but were not always exactly one unit away from the boundary to avoid potential cues to the purpose of the study. Target pairs were not presented together as pairs during the study but were treated as “paired” for design and analysis only.

#### Procedure

Testing took place in a testing room within the university lab space or in a designated area of the science center, and participants received a small toy or other item for participating. After consent procedures, the study began with the experimenter saying, “In this game, you will see a number line from 0 to 100, and I’ll ask you to show me where you think some numbers should go on the line.” Target numerals were printed on 1-inch round labels, presented one at a time at the child’s eye level, and read aloud by the experimenter as presented. Target numerals were presented in this way, rather than appearing on the paper near the response line, to avoid providing spatial cues that could influence responses (e.g., Zax et al., 2019). Participants were asked to estimate the location of each target numeral by drawing a line (in pencil) through the number line. The experimenter said in the early trials, “If 0 goes here [point to left endpoint] and 100 goes here [point to right endpoint], where does [N] go?”. The experimenter did not repeat this statement on each consecutive trial if participants were comfortable with the task, but for each trial the target numeral was held up and read aloud by the experimenter. After the participant responded by drawing a pencil mark through the number line, the researcher placed the target number in the upper right-hand corner and turned the page in the booklet to advance to the next trial. Participants’ marked locations were converted to their corresponding numbers from 0–100 using a custom ruler created for this purpose.

### Results and discussion

Overall error in accuracy was assessed by percent absolute error (PAE), a directionless measure calculated by dividing the absolute difference between the estimated location and the actual location of a numeral by the numerical range, then multiplying by 100 to yield a percentage (Booth & Siegler, 2006; Lai et al., 2018; Slusser et al., 2013). Overall accuracy was consistent with previous reports of performance and of decreasing average percent absolute error with age (Lai et al., 2018; Williams et al., 2022): 5-year-olds’ PAE = 18.10%, 6-year-olds’ PAE = 11.63%, 7-year-olds’ PAE = 10.69%, 8-year-olds’ PAE = 7.68%.

Individual estimates that differed from the group mean for a given target numeral by more than two standard deviations were excluded (3.18% of estimates for 5-year-olds, 5.40% for 6-year-olds, 3.90% for 7-year-olds, 5.88% for 8-year-olds). We used *decade difference scores* as indices of left digit effects. To determine whether estimates were influenced by the leftmost digit, for each participant we calculated decade difference scores for the eight critical pairs of target numerals (one for each pair: 18/22, 29/31, 38/42, 49/51, 59/61, 69/71, 78/82, 88/92). We planned to exclude participants at this point if more than five critical pairs were missing due to outlier removal (*n* = one 8-year-old). We calculated the difference between placements for the paired numerals by first subtracting the placement for the smaller from the placement for the larger (e.g., the placement for 51 minus the placement for 49). We then subtracted the absolute difference between paired numerals (e.g., we subtracted 2 from the difference between the estimates for 51 and 49). Next, we averaged the eight resulting decade difference scores to yield one mean decade difference score for each participant. Decade difference scores significantly greater than zero provide evidence of a left digit effect, indicating that paired numerals were placed too far apart with the larger numeral systematically placed to the right of the smaller numeral.

**Fig. 1:**
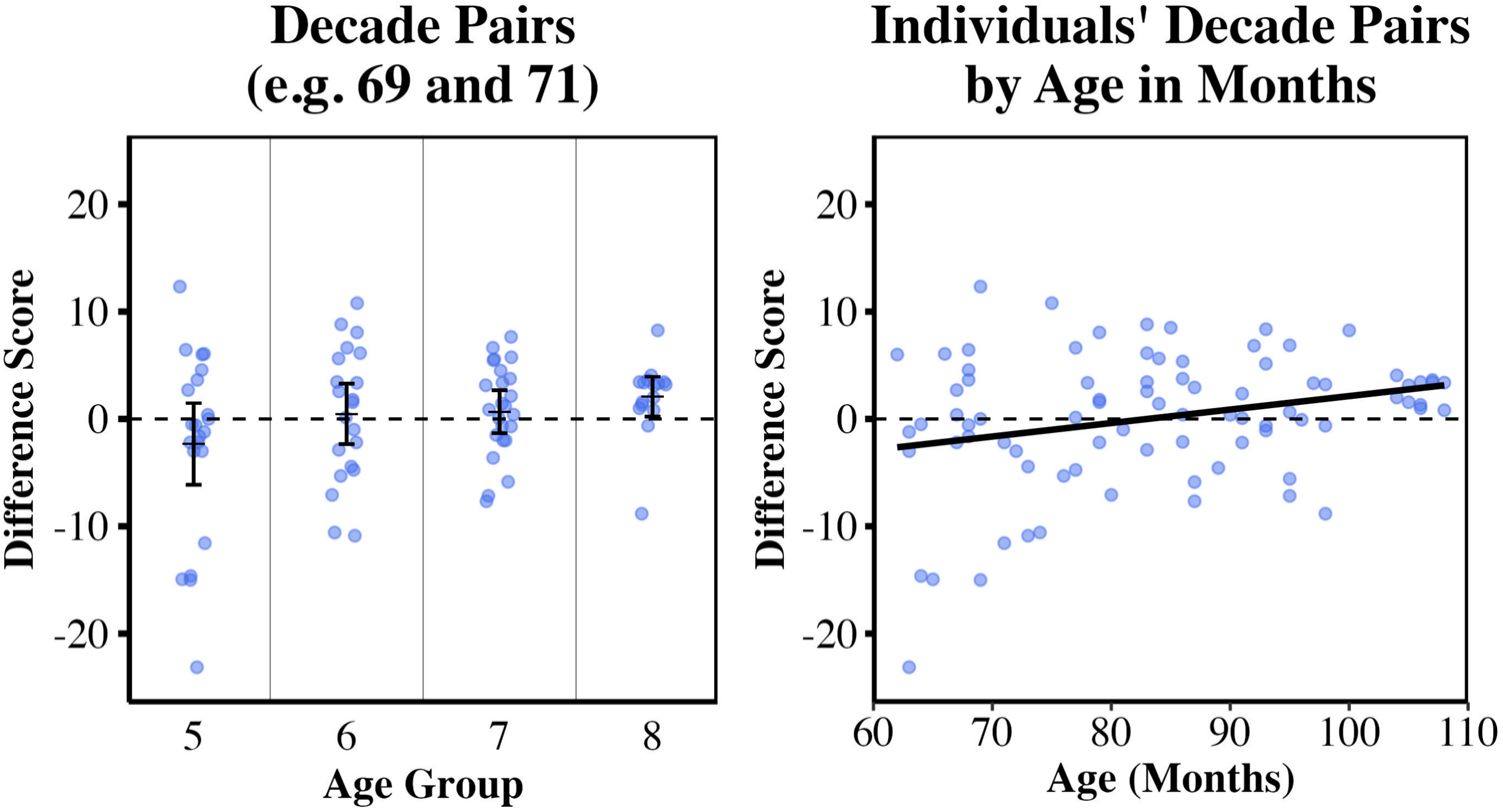
Visualizations of 0–100 left digit bias (decade difference scores) by age. Datapoints represent individual decade difference scores, a measure of left digit bias for the 0–100 range. **Left panel:** Group mean decade difference scores for each age group (in years) and 95% confidence intervals are shown. Mean decade difference scores greater than zero indicate left digit effects. **Right panel:** Individual children’s decade difference scores are plotted with respect to age in months. Left digit bias is correlated with age in months for this age range

To answer our primary question of whether children’s performance on the 0–100 number line task exhibited left digit bias, we asked if decade difference scores were significantly greater than zero for children in each age group (Fig. 1). Because the question of interest here is whether the left digit effect is present or absent in each distinct age group and the corresponding set of tests was planned in advance, we conducted separate *t*-tests within each age group and focus on discussing and depicting results without correction for multiple comparisons. We also provide outcomes with Bonferroni correction for multiple comparisons. Independent single-sample *t*-tests (one-tailed) revealed that decade difference scores were greater than zero for 8-year-olds, *M* = 2.07, *SD* = 3.50; *t*(15) = 2.36, *p* = .016, *d* = 0.59. Decade difference scores were not significantly greater than zero for 5- to 7-year-olds (for age 5: *M* = −2.34, *SD* =8.59; for age 6: *M* = 0.47, SD = 6.20; for age 7: *M* = 0.67, *SD* = 4.75; all *|t|*s < 1.28 all *p*s > .216). (If conservative Bonferroni corrections for multiple comparisons are carried out, alpha = .0125, 8-year-olds’ decade difference scores are not significantly greater than zero.) This finding is consistent with a left digit bias in 8-year-olds’ 0–100 number line placements, but we found no evidence of this bias for younger age groups in this context (Fig. 1). The right panel of Fig. 1 also visualizes this measure of left digit bias with respect to age in months. Left digit bias on the 0–100 task for the children in Study 1, age range 5–8 years, was correlated with age in months, *r* = .271; *p* = .013; this differs from Williams et al. (2022).

Could there be a left digit effect in some of these age groups that we cannot detect due to lack of power? This is possible, but there are at least two reasons to think otherwise: first, left digit bias is often a large effect, and second, the 5-year-olds have negative mean difference scores and the 6- and 7-year-olds have means < 1. To ask whether these data provide positive evidence of a non-effect, we conducted equivalence tests using the TOST procedure (Lakens, 2017; Lakens et al., 2018). We asked specifically whether we could confirm that the decade difference scores did not differ meaningfully from zero (defined here as falling within equivalence bounds −1 to 1). Equivalence tests were not significant for any age group, so we do not have statistical evidence for the claim that there’s no left digit bias in these age groups. Power may be a concern here, as equivalence tests require more power than corresponding null hypothesis testing (Lakens, 2017).

In addition to calculating decade difference scores as the index of the left digit effect, we calculated similar scores for within-decade pairs. These within-decade difference scores used target numeral pairs with the same leftmost digit and magnitudes approximately 2–3 units apart (36/38, 47/49, 71/74, 86/88). As with decade difference scores, we calculated the difference between placements for the larger numerals and placements for the smaller numerals and then subtracted the true difference between target numerals (e.g., the placement for 38 minus the placement for 36 minus 2). The four within-decade difference scores were averaged to yield one mean within-decade difference score for each participant. Within-decade difference scores that are not significantly greater than 0 indicate there’s no evidence that the larger numerals in the pairs were systematically placed too far to the right of the smaller numerals. One-tailed single-sample t-tests showed that within-decade difference scores were not significantly greater than 0 for any age group (5-year-olds: *M* = −0.26, *SD* = 11.70, *t*(21) = −0.10, *p* = .541; 6-year-olds: *M* = −4.34, *SD* = 5.76,* t*(20) = −3.46, *p* = .999; 7-year-olds: *M* = 0.85, *SD* = 5.40, *t*(23) = 0.77,* p* = .225; 8-year-olds: *M* = −0.44, *SD* = 4.29, *t*(15) = −0.41, *p* = .658). There was no evidence of larger numerals in these pairs being placed systematically to the right for any age group. Could there be an effect analogous to left digit bias in these scores that we cannot detect due to lack of power? This is unlikely because for three of four age groups, displacement was in the opposite direction (negative mean difference scores). To ask whether these data provide positive evidence for a null effect, we conducted equivalence tests using the TOST procedure (Lakens, 2017; Lakens et al., 2018), asking whether we could confirm that decade difference scores did not differ meaningfully from zero (defined as falling within equivalence bounds −1 to 1). Equivalence tests were not significant for any age group, so we do not have statistical evidence for the claim that these age groups show no effect. Again, power may be a concern here, as equivalence tests require more power than corresponding null hypothesis testing (Lakens, 2017).

We conducted an exploratory ANOVA with difference score as the dependent measure, within-participants factor pair type (decade difference scores vs. within-decade difference scores), and between-participants factor age group (age in years). We found no main effect of age in years (*F*(3, 79) = 2.64, *p* = .056) or pair type, (*F*(1, 79) = 1.02, *p* = .317). There was no significant interaction between difference score type and age (*F*(3, 79) = 1.52, *p* = .216).

We also computed difference scores for pairs of numerals with the same leftmost (tens) digit but relatively distant magnitudes (e.g., 82 and 88). Because these pairs consist of numerals from the high and low ends of a within-decade range, we refer to them as high-low difference scores (Lai et al., 2018). The purpose is to find out whether children successfully differentiate target numerals that share a leftmost digit but have relatively different magnitudes. High-low difference scores were calculated by subtracting the estimate for the lower numeral from the estimate for the higher numeral (e.g., estimate for 88 minus estimate for 82). For each participant, we calculated one average high-low difference score from eight pairs of numerals: 12/18, 22/29, 31/38, 42/49, 51/59, 61/69, 71/78, 82/88. We planned to exclude participants from this analysis if more than five pairs were missing due to outlier removal, but none required exclusion. If high-low difference scores are not greater than zero, on average (e.g., numerals like 82 and 88 are placed in approximately the same location), this would suggest that participants are relying solely on leftmost (tens place) digits to guide their estimates, and are not differentiating numerals with the same leftmost digits even when they have rather different magnitudes. One-tailed single-sample t-tests found that high-low difference scores were greater than zero for all age groups: 5-year-olds: *M =* 5.89, *SD* = 10.09, *t*(21) = 2.74, *p* =.006, *d* = .58; 6-year-olds: *M* = 2.93, *SD* = 5.38, *t*(20) = 2.49, *p* = .011, *d* = 0.54; 7-year-olds: *M* = 3.84, *SD* = 3.71, *t*(23) = 5.07, *p* < .001, *d* = 1.04; 8-year-olds: *M* = 3.59, *SD* = 3.02, *t*(15) = 4.76, *p* < .001, *d* = 1.19. (Children produced reliably different estimates for lower and higher numerals within the same decade even with correction for multiple comparisons, alpha = .0125.) All age groups thus produced reliably different estimates for the lower and higher numerals within the same decade range; they did not simply place numerals with the same leftmost digits in the same positions.

To summarize these findings, children in all four age groups, 5 through 8 years of age, successfully differentiated numbers like 31 and 38 (same left digits, reasonably different magnitudes), showing that they didn’t rely solely on left digits in this task. No children put numbers like 36 and 38 too far apart (same left digits, very similar magnitudes). Our main test for left digit effects showed that the oldest children placed numbers like 38 and 42 too far apart (different left digit, very similar magnitudes) but we did not find evidence of this in younger children. Additionally, left digit bias was correlated with age in months. Overall, in this 0–100 number range, evidence for left digit bias in children’s paper-and-pencil number line placements was only observed in the oldest children we tested, roughly consistent with findings from other task formats (e.g., Lai et al., 2018; Williams et al., 2022). For the two-digit numbers used in this task, 8-year-olds systematically placed target numerals with the same approximate magnitudes but different tens digits farther apart than would be expected based on their absolute difference. Effect size was moderate for 8-year-olds’ left digit bias in this 0–100 range. As in Williams et al. (2022), there was evidence for the presence of a left digit effect roughly around age 8 years in children’s 0–100 placements as assessed through decade difference scores, and we found no evidence that 5- to 7-year-olds exhibit the effect at the group level. However, we do not have direct statistical evidence for the claim that there is no effect in the younger children whose mean decade difference scores are positive (likely due to power).

We note that when conservative multiple-comparison correction is applied for our planned analyses, 8-year-olds’ decade difference scores are not significantly greater than zero. Williams et al. (2022) reported something similar: for 8-year-olds tested on 0–100 number line estimation, varying analysis choices led to different findings of the presence versus absence of left digit effects as measured by group-level decade difference scores. Other findings and additional analyses may help to contextualize this pattern. First, both 7- and 8-year-olds have shown a large and robust left digit bias in 0–1,000 number line estimation (e.g., Lai et al., 2018), so the bias is clearly present by age 8 years in some numerical ranges and task formats. Second, slightly older children showed a strong left digit bias for the same 0–100 range in an electronic format, while children under 8 years of age did not show evidence of the bias (Williams et al, 2022), suggesting that left digit effects in the 0–100 range arise roughly around this age in a typical US educational context (even if not robustly observed at age 8 years for a 0–100 range). Third, Williams et al. (2022) reported that 76% (16/21) of individual 8-year-olds in their sample had positive decade difference scores. In the present Study 1, 88% (14/17) of individual 8-year-olds had positive decade difference scores, compared with 41% of 5-year-olds, 57% of 6-year-olds, and 63% of 7-year-olds. In both these 0–100 studies, 8-year-olds’ individual decade difference scores are positive significantly more frequently than would be expected by chance when subjected to post-hoc sign tests. Collectively, the evidence to date suggests that left digit bias is emerging roughly in the range of 7–8 years of age (at least in these populations). Visual inspection of the dot plots in Fig. 1 also suggests this is the case, and that it is possible larger sample sizes would have revealed significant left digit bias around age 7 years. In contrast, there appears to be little to no evidence of the bias emerging any earlier, at the group level. Study 2 extends the paper-based task format to a larger age range and number range, while including children that overlap with the age range tested in Study 1.

## Study 2

This study investigated children’s left digit bias in a paper-based number line estimation task with a larger numerical range. Children in this study ranged from 7 to 11 years old and were presented with a 0–1,000 number line on paper, to align with previous research on left digit effects using electronic devices (Lai et al., 2018). In Lai et al. (2018), children in this age range showed large left digit effects on computer- and tablet-based speeded and non-speeded 0–1,000 tasks, but it is unknown whether these findings extend to a paper version of the task. Accordingly, we asked if 7- to 11-year-olds’ numerical placements are influenced by the particular hundreds digits that instantiate three-digit target numerals in a 0–1,000 paper-based number line task.

### Methods

#### Participants

One hundred and nineteen children were recruited from a university participant database and a local science center. Six children were initially excluded for noncompliance (one 7-year-old, two 8-year-olds, and one 10-year-old), producing estimates uncorrelated with target numbers (one 8-year-old), or experimenter error (one 8-year-old). After these exclusions, our sample included 22 7-year-olds (*M*_*age*_ = 7;5, range = 7;0 – 7;11, 13 female, nine male), 21 8-year-olds (*M*_*age*_ = 8;6, range = 8;1 – 8;11, 14 female, seven male), 24 9-year-olds (*M*_*age*_ = 9;6, range = 9;0 – 9;11, 13 female, 11 male), 24 10-year-olds (*M*_*age*_ = 10;6, range = 10;0 – 10;10, 10 female, 13 male), and 22 11-year-olds (*M*_*age*_ = 11;5, range = 11;0 – 11;11, 15 female, seven male).

#### Stimuli

Stimuli were like those used in Study 1, except that the numerical range used for the number line task was 0–1,000. Participants completed one block of 38 trials, with the following target numerals presented in a different random order to each participant: 47, 51, 98, 102, 147, 153, 199, 202, 249, 252, 298, 302, 349, 351, 398, 403, 449, 453, 499, 502, 547, 552, 597, 601, 647, 652, 699, 703, 747, 753, 798, 802, 848, 853, 899, 901, 949, 953. Eight critical pairs of target numerals, falling on either side of hundreds boundaries, were embedded within this set to assess left digit effects: 199/202, 298/302, 398/403, 499/502, 597/601, 699/703, 798/802, 899/901.

#### Procedure

The procedure was the same as that used for Study 1 except that the experimenter began by saying, “In this game, you will see a number line from 0 to 1,000 and I’ll ask you to show me where you think some numbers should go on the line.” Also, to begin a trial, the experimenter turned to a new page in the booklet and said, “If 0 goes here [point to left endpoint] and 1,000 goes here [point to right endpoint], where does [N] go?”. The experimenter did not repeat this statement on each consecutive trial once participants were comfortable with the task, but the target numeral was held up and read aloud by the experimenter for each trial.

### Results and discussion

Overall accuracy error was consistent with previous reports of performance and decreasing average percent absolute error with age (Lai et al., 2018; Williams et al., 2022): 7-year-olds’ PAE = 14.02%, 8-year-olds’ PAE = 11.23%, 9-year-olds’ PAE = 7.20%, 10-year-olds’ PAE = 5.90%, and 11-year-olds’ PAE = 5.15%.

Individual estimates were excluded if they differed from the group mean for a given target numeral by more than 2 standard deviations (3.71% of estimates for 7-year-olds, 4.55% for 8-year-olds, 6.03% for 9-year-olds, 4.71% for 10-year-olds, 4.55% for 11-year-olds). We used hundreds difference scores as an index of the left digit effect. We first calculated individual hundreds difference scores.^1^ For each participant, we calculated *placement for larger numeral – placement for smaller numeral – absolute difference between numerals* (e.g., the placement for 302 minus the placement for 298 minus 4) for eight critical pairs of target numerals: 199/202, 298/302, 398/403, 499/502, 597/601, 699/703, 798/802, 899/901. Next, we averaged the eight individual hundreds difference scores to yield one average hundreds difference score for each participant. We planned to exclude individuals’ data at this point if five or more critical pairs were missing due to outlier removal (*n* = one 10-year-old). Hundreds difference scores greater than zero indicate that paired numerals were placed too far apart, with the larger numeral systematically placed to the right; this is one form of evidence of a left digit effect.

**Fig. 2:**
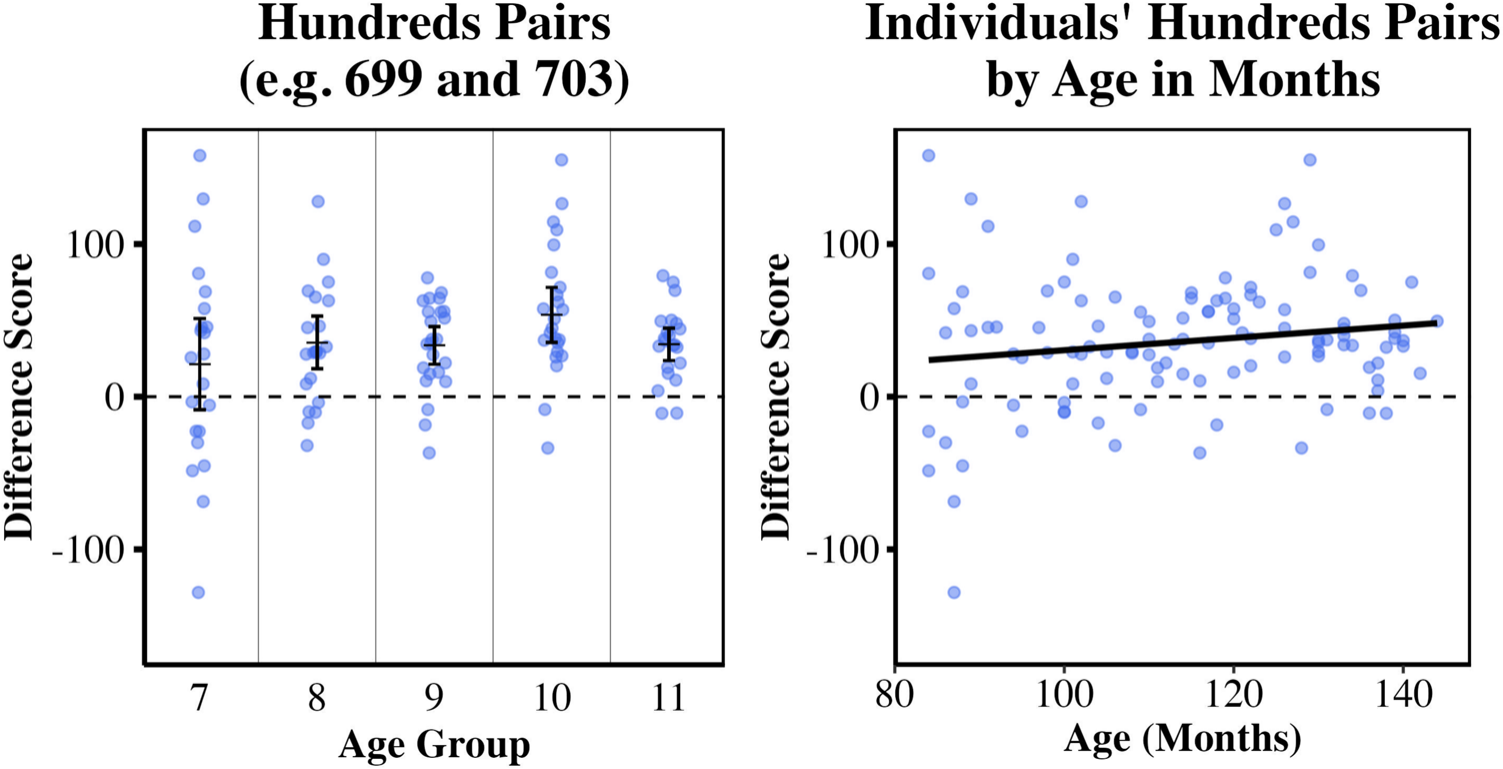
Visualizations of 0–1,000 left digit bias (hundreds difference scores) by age. Datapoints represent individual hundreds difference scores, a measure of left digit bias for the 0–1,000 range. **Left panel:** Group mean hundreds difference scores for each age group (in years), and 95% confidence intervals are shown. Mean decade difference scores greater than zero indicate left digit effects. **Right panel:** Individual children’s hundreds difference scores are plotted with respect to age in months. Left digit bias is not correlated with age in months for this age range

Single-sample one-tailed *t*-tests revealed that hundreds difference scores were significantly greater than zero for children ages 8, 9, 10, and 11, with large effect sizes (8-year-olds: *M* = 35.49, *SD* = 37.79, *t*(20) = 4.30, *p* < .001, *d* = 0.94; 9-year-olds: *M* = 33.60, *SD* = 29.17, *t*(23) = 5.64, *p* < .001, *d* = 1.15; 10-year-olds: *M* = 53.59, *SD* = 41.65, *t*(22) = 6.17, *p* < .001, *d* = 1.29; 11-year-olds: *M* = 34.53, *SD =* 24.01, *t*(21) = 6.71, *p* < .001, *d* = 1.43). These findings for ages 8–11 years remained significant following correction for multiple comparisons, alpha = .01. Hundreds difference scores were not significantly greater than zero for 7-year-olds (*M* = 21.33, *SD* = 67.47, *p* = .076); however, there is also no evidence for a positive claim that these 7-year-olds have no left digit bias (either by observation of the mean score or by formal equivalence test). See Fig. 2 for participants’ average hundreds difference scores. The right panel of Fig. 2 also visualizes this measure of left digit bias with respect to age in months. Left digit bias on the 0–1,000 task for these children, age range 7–11 years, was uncorrelated with age in months, *p* > .05; this is consistent with Lai et al. (2018).

We also calculated fifties difference scores for target numerals around fifties boundaries: 147/153, 249/252, 349/351, 449/453, 547/552, 647/652, 747/753, 848/853, 949/953. As with hundreds difference scores, we calculated the difference between placements for the larger target numeral and the smaller target numeral in each pair and subtracted the absolute difference between numerals (e.g., placement for 153 minus placement for 149 minus 4). The nine fifties difference scores were averaged to yield a mean fifties difference score for each participant. Fifties difference pairs, like hundreds difference pairs, have similar magnitudes, but fifties difference pairs share leftmost digits. Similar to the within-decade scores of Study 1, fifties scores are a control to evaluate whether participants might also place other pairs of numerals with similar magnitudes too far apart on a number line. One-tailed single-sample t-tests show that fifties difference scores were not greater than zero for any age group (7-year-olds: *M* = 8.98, *SD* = 52.32,* t*(21) = 0.81, *p* = .215; 8-year-olds: *M* = −15.50, *SD* = 39.50, *t*(20) = −1.80, *p* = .956; 9-year-olds: *M* = −1.911, *SD* = 28.03, *t*(23) = −0.33, *p* = .629; 10-year-olds: *M* = −7.69, *SD* = 16.77,* t*(22) = −2.20,* p* = .981; 11-year-olds: *M* = −11.14, *SD* = 19.16, *t*(21) = −2.73, *p* = .994). There was no evidence of the larger numerals in these fifties pairs being placed systematically to the right for any age group. This finding reveals that although the left digit effect appears for target numeral pairs for which leftmost digits differ (like 302 and 298), there is no evidence of an analogous effect for similar pairs that share leftmost digits (like 351 and 349). It is unlikely that we simply failed to find such an effect due to lack of power, because for four of five age groups the mean displacement was in the opposite direction (negative mean difference scores). To ask whether the data provided positive evidence for a non-effect for these fifties scores, we conducted equivalence tests using the TOST procedure (Lakens, 2017; Lakens et al., 2018). We asked specifically whether we could confirm that the fifties scores did not differ meaningfully from zero (defined as falling within equivalence bounds −10 to 10). Equivalence tests were not significant for any age group, so we do not have statistical evidence to claim that fifties scores are not meaningfully different from zero (with four of the five scores less than zero). Power could be a concern here, as equivalence tests require more power than corresponding null hypothesis testing (Lakens, 2017).

We conducted an ANOVA with difference score as the dependent measure, within-participants factor pair type (hundreds difference scores vs. fifties difference scores), and between-participants factor age group (age in years). We found no main effect of age in years (*F*(4, 107) = 0.88, *p* = .477), but there was a main effect of pair type (*F*(1, 107) = 56.63, *p* < .001, η^2^ = 0.22), consistent with the t-test results showing that hundreds scores were strongly positive and fifties scores were near zero. There was no interaction between pair type and age group (*F*(4, 107) = 2.31, *p* = .063).

We again tested whether children in each age group reliably differentiated numerals with identical leftmost digits that were located at the higher and lower ends of their range. We computed difference scores for pairs of numerals with the same leftmost digit but very different magnitudes (e.g., 802 and 899, referred to as high-low difference scores). For each participant, we calculated one average high-low difference score from eight pairs of numerals: 102/199, 202/298, 302/398, 403/499, 502/597, 601/699, 703/798, 802/899. We planned to exclude participants if more than five pairs were missing due to outlier removal, but no participants required exclusion. High-low difference scores greater than zero indicate that children did differentiate these numbers rather than simply giving the same response on average for all target numerals with the same leftmost digit. High-low difference scores were greater than zero for children ages 7–11 (7-year-olds: *M* = 45.82, *SD* = 49.90, *t*(21) = 4.31, *p* < .001, *d* = .92; 8-year-olds: *M* = 33.54, *SD* = 38.62, *t*(20) = 3.98, *p* < .001, *d* = .87; 9-year-olds: *M* = 46.37, *SD* = 36.55, *t*(23) = 6.21, *p* < .001, *d* = 1.27; 10-year-olds: *M* = 36.55, *SD* = 40.77, *t*(22) = 4.30, *p* < .001, *d* = .90; 11-year-olds: *M* = 50.29, *SD* = 28.43, *t*(21) = 8.30, *p* < .001, *d* = 1.77). (High-low difference scores were also greater than zero for all ages following correction for multiple comparisons, alpha = .01.) Thus, all age groups produced reliably different estimates for numerals like 802 and 899 and we observed no evidence that some young children relied solely on leftmost digits when performing this task. This differs from a previous finding (Lai et al., 2018); we suggest the previous observation does not represent a reliable developmental pattern.

**Fig. 3:**
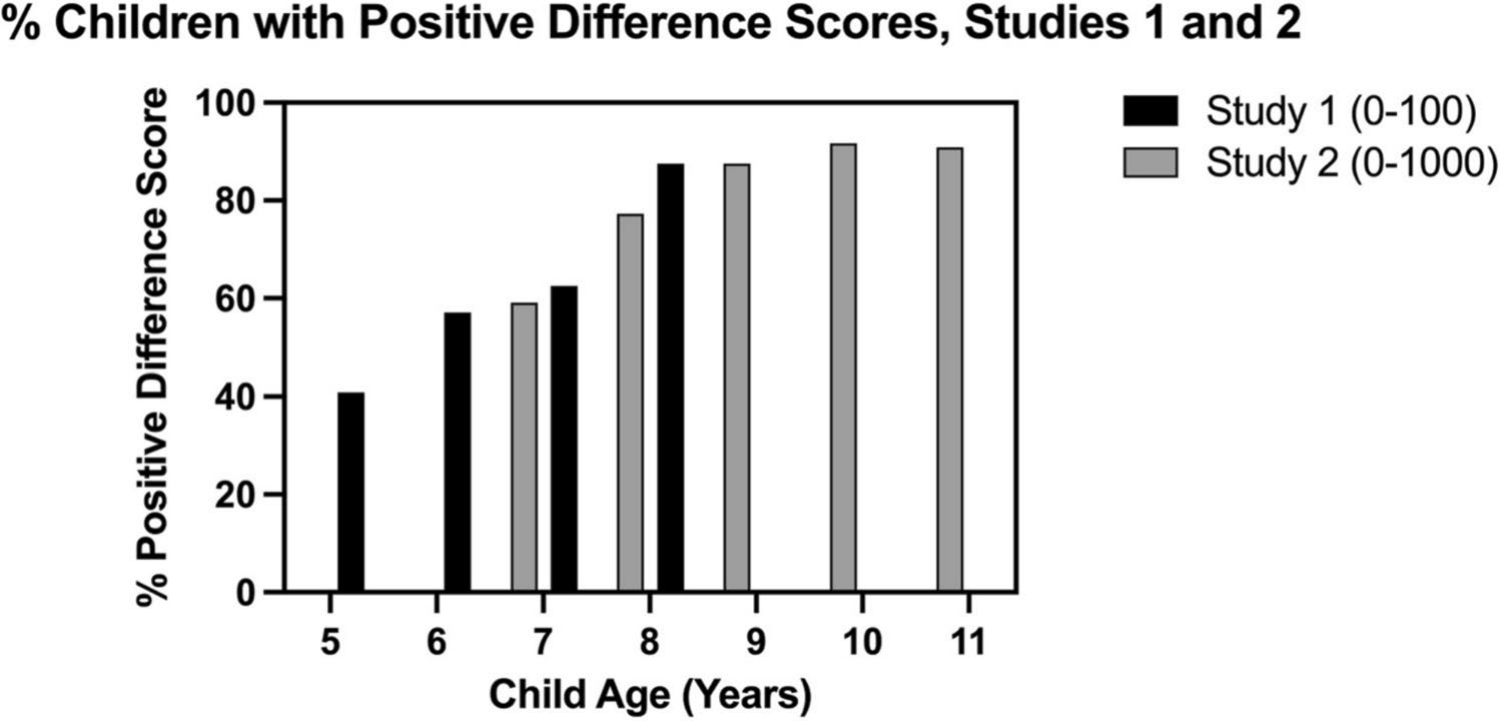
Percentages of children with individual decade difference scores (Study 1) or hundreds difference scores (Study 2) that were positive, consistent with the sign of a left digit bias. These percentages do not reflect significant differences from zero, but simply scores that were positive (rather than being equal to zero or negative)

In Study 2, we asked whether the left digit effect is observed in children aged 7–11 years on a paper version of a standard number line estimation task with a larger numerical range. In line with Lai et al. (2018), 8- to 11-year-olds showed large left digit effects on this 0–1,000 task, placing numerals like 798 and 802 significantly farther apart than would be expected based on their magnitudes, with the larger numeral placed systematically to the right. We found no evidence for the same effect in 7-year-olds here, in contrast to some findings of Lai et al. (2018); in that work, 7-year-olds showed a left digit effect in a non-speeded computer-based task but not in a speeded computer-based task. However, in the present study, it is likely that left digit bias is beginning to emerge in the 7-year-old group. Their group mean hundreds difference score was > 20 units, and 64% of individual 7-year-olds (*n* = 14) had positive hundreds difference scores (as did 81% of 8-year-olds, *n* = 17; 88% of 9-year-olds, *n* = 21; 91% of 10-year-olds, *n* = 21; and 91% of 11-year-olds, *n* = 20). Figure 3 depicts the percentages of individuals in each age group with positive decade or hundreds difference scores for Studies 1 and 2. Post hoc exploratory analysis of the 7-year-olds’ data further showed that their hundreds difference scores were strongly negatively correlated with overall accuracy error (*r* = -.618, *p* = .002). The more accurate half of the 7-year-olds nearly all had positive hundreds difference scores (consistent with left digit bias) while the less accurate half nearly all had negative scores (inconsistent with left digit bias). This finding also argues for emerging left digit bias in this age range.

The results of Study 2, taken together, extend the findings of Study 1 to show that 8- to 11-year-olds display a robust left digit effect on paper-based number line estimation with a larger numerical range (0–1,000), while the 7-year-olds did not exhibit a significant effect at the group level. Study 3 uses the tasks of both Study 1 and Study 2 in adult participants.

## Study 3

In Study 3, adults completed standard 0–100 and 0–1,000 number line tasks on paper in two separate lab visits. Adults have previously shown a left digit effect in 0–100 number line estimation (Patalano et al., 2023; Williams et al., 2020), including a single study using paper and pencil (Savelkouls et al., 2020) and in electronic 0–1,000 tasks (e.g., Lai et al., 2018; Williams et al. 2023). Findings of a left digit effect in the 0–1,000 range have not been extended to adults’ performance on a paper version of the number line task. This study was designed in parallel with a corresponding electronically-administered 0–100 number line task that was published previously (Experiment 2, Williams at al., 2022), in addition to conceptually replicating adult findings from Lai et al. (2018). We asked whether adults’ estimates were overly influenced by tens digits in 0–100 paper-based number line tasks and by hundreds digits in 0–1,000 paper-based tasks.

### Methods

#### Participants

Twenty-four adults were recruited from a university participant pool. One adult did not complete the 0–1,000 task and was excluded. Our final sample included 23 adults (*M*_*age*_ = 18.8 years, range = 18–22, nine female, 14 male).

#### Stimuli

Stimuli were identical to those used in Studies 1 and 2. In the 0–100 task, participants completed one block of 30 trials, and in the 0–1,000 task, participants completed one block of 38 trials. The target numerals for each number line task were identical to Studies 1 and 2, and once again, were presented in a different random order to each participant. The same eight critical target numeral pairs from Studies 1 and 2 were used to assess left digit effects.

#### Procedure

The procedure was generally the same as that used for Studies 1 and 2 except that participants completed both 0–100 and 0–1,000 tasks, in two separate sessions approximately 2 weeks apart (*M* = 13.9 days, range = 7–21 days). Order was counterbalanced (11 participants completed 0–100 first, 12 participants completed 0–1,000 first). For each session, the experimenter began by saying, “In this game, you will see a number line from 0 to 100 [or 0 to 1,000] and I’ll ask you to show me where you think some numbers should go on the line.”

### Results and discussion

**Fig. 4:**
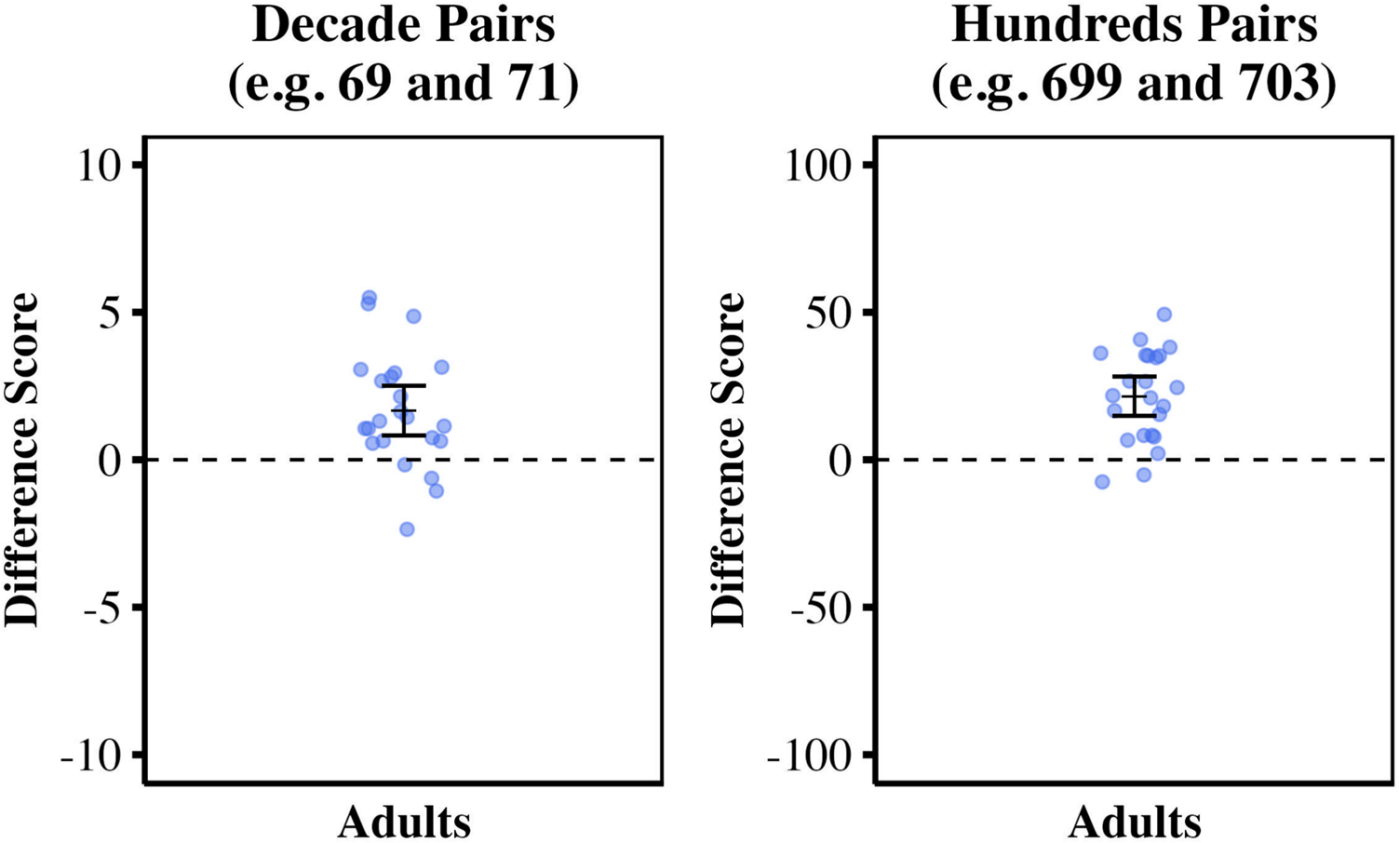
Adults’ mean difference scores for 0–100 and 0–1,000 number lines. Decade difference scores (**left**) and hundreds difference scores (**right**) are shown for each number line task. Difference scores greater than zero indicate left digit effects. Group means and 95% confidence intervals are shown

Overall accuracy error was consistent with previous reports (Lai et al., 2018; Williams et al., 2022): adults’ 0–100 PAE = 3.22% and 0–1,000 PAE = 4.1%. For both the 0–100 and 0–1,000 number line, we planned to exclude individual estimates that differed from the group mean for a given target numeral by more than 2 standard deviations, but no estimates required exclusion. As in Studies 1 and 2, to determine whether estimates were influenced by the left digit effect, we calculated individual decade and hundreds difference scores for the 0–100 and 0–1,000 tasks respectively. We averaged the eight individual difference scores for each number line to yield one average decade difference score and one average hundreds difference score for each participant. We planned to exclude individuals’ data at this point if five or more pairs were missing due to outlier removal, but none required exclusion. As in Studies 1 and 2, decade difference scores and hundreds difference scores greater than zero indicate that the larger numeral in each critical pair was systematically placed to the right of the smaller, evidence for the left digit effect. Adults’ decade difference scores were significantly greater than zero, indicating a large left digit effect for two-digit numerals (*M* = 1.67, *SD* = 1.95, *t*(22) = 4.10, *p* < .001, *d* = 0.86). Adults’ hundreds difference scores were significantly greater than zero, indicating a very large left digit effect for three-digit numerals (*M* = 21.56, *SD* = 15.37), *t*(22) = 6.73, *p* < .001, *d* = 1.40). See Fig. 4 for adults’ left digit bias for both tasks.

As in Studies 1 and 2, we also calculated within-decade difference scores for the 0–100 number line and fifties difference scores for the 0–1,000 number line, to serve as controls. Participants’ within-decade difference scores, calculated from the same 4 numeral pairs used in Study 1, were not significantly greater than zero (*M* = −1.09, *SD* = 2.23, *t*(22) = −2.34, *p* = .986). Fifties difference scores were also not significantly greater than 0 (*M* = −4.10, SD = 13.36, *t*(22) = −1.47, *p = .922*). This indicates that for the 0–100 and 0–1,000 number lines, left digit effects were seen for numbers with very similar magnitudes and different leftmost digits, but there was no evidence of analogous effects for other kinds of pairs a few units apart that shared leftmost digits. We directly compared the within-decade to the decade scores for the 0–100 range and the fifties to the hundreds scores for the 0–1,000 range. Decade difference scores were significantly greater than within decade difference scores (*t*(22) = 4.81, *p* < .001,* d* = 1.00) and hundreds difference scores were significantly greater than fifties difference scores (*t*(22) = 5.85, *p* < .001, *d* = 1.22). Mean within-decade and fifties scores were both negative, so there is no reason to suspect a small but undetected effect. Further, equivalence tests support the claim that fifties difference scores (but not within-decade difference scores) are not meaningfully different from zero (TOST with equivalence bounds −10 to 10, lower bound *t*(22) = −5.06; *p*_*lower*_ < .001, upper bound *t*(22) = 2.12, *p*_*upper*_ = .023).

We investigated whether estimates were exclusively influenced by leftmost digits, as in the previous studies with children. We computed high-low difference scores for pairs of numerals with the same leftmost digit but different magnitudes (e.g., 22 and 29 for the 0–100 number line and 202 and 298 for the 0–1,000 number line). For each participant, we calculated an average high-low difference score for the 0–100 number line and an average high-low difference score for the 0–1,000 number line, using the same eight pairs of numerals for each number line from Studies 1 and 2. We planned to exclude participants if more than five pairs were missing due to outlier removal, but none required exclusion. For the 0–100 number line, adults’ high-low difference scores were significantly greater than zero (*M* = 5.41, *SD* = 2.16, *t*(22) = 11.98, *p* < .001, *d* = 2.50), indicating that adults were able to reliably differentiate lower and higher numerals within the same decade (e.g., 22 and 29). For the 0–1,000 number line, adults’ high-low difference scores were greater than zero (*M* = 72.71, *SD* = 17.94; *t*(22) = 19.44, *p* < .001, *d* = 4.05), indicating that adults reliably differentiate lower and higher numerals within the same hundreds range (e.g., 802 and 899). On both the 0–100 and 0–1,000 number lines, adults were able to reliably differentiate numerals with identical leftmost digits but significantly different magnitudes, showing that, as expected, they do not base estimates exclusively on leftmost digits.

In Study 3, we asked whether the left digit effect is evident in adults’ performance on a paper version of two number line tasks, using a 0–100 range and a 0–1,000 range. Adults showed a large left digit effect for both ranges, estimating numerals like 88 and 92 on the 0–100 number line and 798 and 802 on the 0–1,000 number line to be significantly further apart than would be expected based solely on their magnitudes, with the larger numeral placed systematically to the right. There was no evidence of analogous effects for nearby pairs of numbers that shared left digits, either for within-decade pairs or fifties pairs; there was positive evidence that no analogous effect was present for fifties pairs. Difference scores for numerals with the same left digit but different magnitudes (e.g., 802 and 899) revealed that unsurprisingly, adults’ placements were not solely determined by leftmost digits in either numerical range: numerals with the same leftmost digits but considerably different magnitudes were reliably differentiated. These results extend the findings of Studies 1 and 2 to show that adults display a left digit effect on paper-based number line estimation with 0–100 and 0–1,000 numerical ranges, consistent with findings from number line estimation tasks in other contexts (Lai et al., 2018; Savelkouls et al., 2020; Williams et al., 2022, 2023).

## Study 4

In Study 4, we directly compared paper and computer task formats in a formally preregistered pair of experiments with a new sample of children, within the age range that reliably exhibits strong left digit bias, and a new sample of adults. Each participant completed both a paper-based and a computer-based 0–1,000 number line estimation task (blocked). Adults exhibit large left digit effects in 0–1,000 number line tasks for both computer-based (Lai et al., 2018; Patalano et al., 2023; Williams et al., 2020; Williams et al., 2023) and paper-based (Savelkouls et al., 2020) formats. Children, at least those over age 8 years, reliably show the same strong biases in 0–1,000 tasks in a computer-based format (Lai et al., 2018; Williams et al., 2022), and the present Study 2 found similar evidence in a paper task. However, a direct comparison of left digit bias across formats has not yet been made. Here, we asked if 0–1,000 number line placements of children (aged 9–12 years) and adults were overly influenced by hundreds digits on both paper-based and computer-based tasks, and if so, whether this left digit bias differed across task formats.

### Methods

#### Participants

Forty-eight children were recruited from a university participant database and from the community. Forty-six adults were recruited on the university campus, including some parents of child participants. Participants whose estimates were uncorrelated with target numerals or who had > 50% outliers in their estimates for either condition were excluded entirely (three adults, one child). Individual target numeral estimates for each participant were excluded if they were more than two standard deviations from the mean for that target. One additional child was excluded due to computer failure, and one additional adult was excluded due to experimenter error. Occasionally, participants failed to respond to a single trial (i.e., if they accidentally turned two pages of the paper booklet at once). These participants were included. No participants were excluded for failure to complete the task*.* After these exclusions, the samples consisted of forty-six children (eight 9-year-olds: *M*_*age*_ = 9;3, range = 9;0 – 9;9, five female, three male; 12 10-year-olds: *M*_*age*_ = 10;4, range = 10;0 – 10;11, eight female, four male; 22 11-year-olds: *M*_*age*_ = 11;5, range = 11;0 – 11;11, ten female, 11 male, 1 nonbinary; four 12-year-olds: *M*_*age*_ = 12;4, range = 12;0 – 12;9, three female, one male) and 42 adults (*M*_*age*_ = 29.2, range = 18–48, 32 female, ten male).

Participants who were missing more than three critical pairs due to outlier exclusions from either condition were excluded at a later stage from left digit effect analyses (one child, two adults)*.* After those exclusions from left digit analyses, the sample included 45 children and 40 adults.

#### Stimuli

For each of the two task formats, participants completed a 0–1,000 number line task in a block of 40 trials with the following target numerals presented in a different, random order to each participant: 199, 202, 218, 249, 252, 298, 301, 327, 348, 351, 398, 402, 435, 447, 451, 474, 499, 502, 532, 549, 552, 588, 597, 601, 649, 652, 664, 699, 703, 749, 751, 768, 798, 802, 812, 847, 851, 873, 899, 901. Eight critical pairs of target numerals, falling on either side of hundreds boundaries, were embedded within this set to assess left digit effects: 199/202, 298/301, 398/402, 499/502, 597/601, 699/703, 798/802, 899/901. Also included were seven pairs on either side of the fifties boundaries used as controls (249/252, 348/351, 447/451, 549/552, 649/652, 749/751, 847/851), and ten filler numerals (218, 327, 435, 474, 532, 588, 664, 768, 812, 873).

For the paper task, stimuli were similar to those used in Study 2 with a numerical range of 0–1,000 and response lines of approximately 24.5 cm. Target numerals were printed in Sans-Serif font on each sheet of paper centered above the number line. For the computer task, participants saw similar stimuli displayed on a lab computer. These stimuli were created using lab.js (labjs.org; Henninger et al., 2019) and presented via Qualtrics software. Each number line was 20 cm long with 1-cm vertical lines at the endpoints, labeled 0 on the left and 1,000 on the right. Target numerals were identical in both tasks, and each participant was presented with the target numerals in a random order (differing across people and across task formats). After a participant clicked the location on the line where they thought the number should go, a red 1cm vertical line appeared on the clicked location and a button labeled “Next” appeared below the number line that could be clicked to move on to the next trial.

#### Procedure

Participants were assigned to complete the paper or the computer task first, with order counterbalanced. The paper task procedure was as in Study 2, except the experimenter did not hold up and read aloud each target numeral, as they were printed on each page. Following consent procedures, the first trial of the study began with the experimenter pointing to each endpoint and saying, “If 0 goes here and 1,000 goes here, where does X (target numeral) go?” This was repeated for one to two more trials and then discontinued. Participants were instructed not to use physical implements to measure (such as their finger or the pen) and not to change their answers after marking a location. After participants drew a pen mark through the number line, they turned the page themselves to move on to the next trial. After every ten trials participants saw a page giving mildly positive feedback about their progress. For the computer tasks, participants were provided with the same verbal instructions used in Study 2 and the current paper task, as well as instructions on the screen introducing the specifics of the computer task. Following every ten trials, positive feedback pages appeared as in the paper task. The experimenter remained in the room with the participant while they completed the automated computer-based task. After participants finished the first task, they were brought into a separate laboratory room to complete the second version. Between the paper and computer blocks, child participants had a short break and chose a sticker to keep.

### Results and discussion

Analyses were formally preregistered (https://aspredicted.org/WQC_ZHW) unless otherwise indicated. We preregistered child and adult analyses as separate experiments but present them together for brevity.

Overall accuracy error was consistent with previous reports (Lai et al., 2018; Williams et al., 2022): children’s PAE_paper_ = 5.1%, children’s PAE_computer_ = 5.2%, adults’ PAE_paper_ = 2.8%, and adults’ PAE_computer_ = 4.0%).^2^ For each age group, a repeated-measures ANOVA was conducted on overall accuracy error (PAE) with within-participants factor task format (paper vs. computer) and between-participants factor order (paper first vs. computer first). No main effect of task format or order and no interaction were found for children (task format:* F*(1,44) = 0.25, *p* = .622; order: *F*(1,44) = 1.06, *p* = .309; interaction: *F*(1,44) = 3.89, *p* = .055) or adults (task format: *F*(1,40) = 0.97, *p* = .330; order: *F*(1,40) = 0.82, *p* = .370; interaction: *F*(1,40) = 1.03, *p* = .317).

**Fig. 5:**
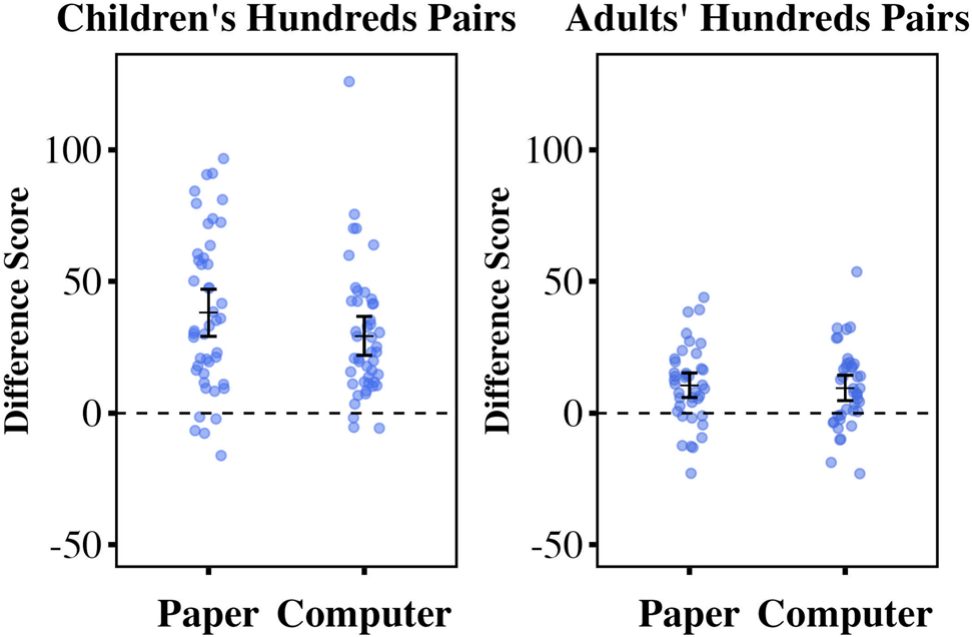
Children’s and adults’ mean hundreds difference scores on 0–1,000 number line tasks. Scores for each task format and age group are shown. Hundreds difference scores greater than zero indicate left digit effects. Mean hundreds difference scores and 95% confidence intervals are shown

As in Studies 2 and 3, left digit effect analyses were conducted on hundreds difference scores; we calculated two hundreds difference scores per participant (paper and computer). Single-sample one-tailed *t*-tests revealed that hundreds difference scores were greater than zero for both children and adults on both the paper and computer tasks (children’s paper: *M* = 38.12, *SD* = 29.63, *t*(44) = 8.63, *p* < .001, *d* = 1.29; children’s computer: *M* = 28.95, *SD* = 24.89, *t*(44) = 7.80 *p* < .001, *d* = 1.16; adults’ paper: *M* = 10.63, *SD* = 14.67, *t*(39) = 4.58, *p* < .001, *d* = 0.73; adults’ computer: *M* = 9.59, *SD* = 15.18, *t*(39) = 3.99, *p* < .001, *d* = 0.63; see Fig. 5). These findings indicate large left digit effects for both formats in both age groups.

For each age group, as preregistered, a repeated-measures ANOVA was conducted on hundreds difference scores using within-participants factor task format and between-participants factor order. For children, there was a significant main effect of task format (*F*(1, 43) = 4.18, *p* = .047, η^2^= 0.03): children’s left digit bias was larger for the paper task than the computer task. There was no main effect of order (*F*(1,43) = 6.59, *p* = .432) and no significant interaction (*F*(1, 43) = 0.04, *p* = .851). For adults, there was no main effect of task format (*F*(1, 38) = 0.17, *p* = .686) or order (*F*(1, 38) = 0.78, *p* = .383). We found a significant interaction between task type and order for adults (*F*(1, 38) = 5.27, *p* = .027, η^2^= 0.04). There was a larger left digit effect for the paper task in adults who completed it first (*M* = 15.27, *SD* = 11.74) compared to those who completed it second (*M* = 5.98, *SD* = 16.08).

We calculated fifties difference scores to serve as controls, also as in previous studies. These were not significantly greater than zero for either task for children (paper: *M* = 1.37, *SD* = 23.16, *t*(44) = 0.40, *p* = .347; computer: *M* = 0.88, *SD* = 27.31, *t*(44) = 0.22, *p* = .415) or adults (paper: *M* = −1.88, *SD* = 13.69, *t*(40) = −0.88, *p* = .808; computer: *M* = −3.49, *SD* = 12.81,* t*(41) = −1.77, *p* = .958). This is consistent with findings from Studies 2 and 3 and prior work; the differential placements used as indices of left digit bias are seen for pairs of numerals with similar magnitudes but differing leftmost digits, but there are no analogous differential placements for pairs with similar magnitudes that share leftmost digits. As in previous studies, we applied the TOST procedure to test for equivalence between each age groups’ fifties difference score for each task format and 0, with equivalence bounds set at −10 and 10. All tests were significant in this case (Adults/Paper: lower bound* t*(40) = −5.56, *p*_*lower*_ < .001, upper bound *t*(40) = 3.80, *p*_*upper*_ < .001; Adults/Computer: lower bound* t*(41) = −6.83, *p*_*lower*_ < .001, upper bound* t*(41) = 3.29, *p*_*upper*_ = .001; Children/Paper: lower bound* t*(43) = −2.38, *p*_*lower*_ =.011, upper bound *t*(45) = 3.30, *p*_*upper*_ < .001; Children/Computer: lower bound* t*(43) = −2.50, *p*_*lower*_ = .008, upper bound *t*(43) = 2.20, *p*_*upper*_ = .017). These results support the positive claim that fifties scores were not meaningfully different from 0.

We again computed high-low difference scores for pairs of numerals with the same leftmost digit, but different magnitudes. For each participant, we calculated an average high-low difference score, using seven pairs of numerals (202/298, 301/398, 402/499, 503/597, 601/699, 703/798, 802/899). One child participant and one adult participant were removed from analyses because they had more than three high-low pairs missing for both the paper and computer formats. Children’s high-low difference scores were significantly greater than zero for both paper (*M* = 58.23, *SD* = 29.76, *t*(44) = 13.12, *p* < .001, *d* = 1.96) and computer (*M* = 58.84, *SD* = 22.94, *t*(44) = 17.21, *p* < .001, *d* = 2.57). Adults’ high-low difference scores were also significantly greater than zero for both paper (*M* = 86.37, SD = 17.02, *t*(40) = 32.49, *p* < .001, *d* = 5.07) and computer (*M* = 86.00, *SD* = 16.74, *t*(40) = 32.90, *p* < .001, *d* = 5.14). As expected, both adults and children reliably differentiated much lower and much higher numerals within the same hundreds range, showing that they do not base their estimates exclusively on a target numeral’s leftmost digit.

**Fig. 6:**
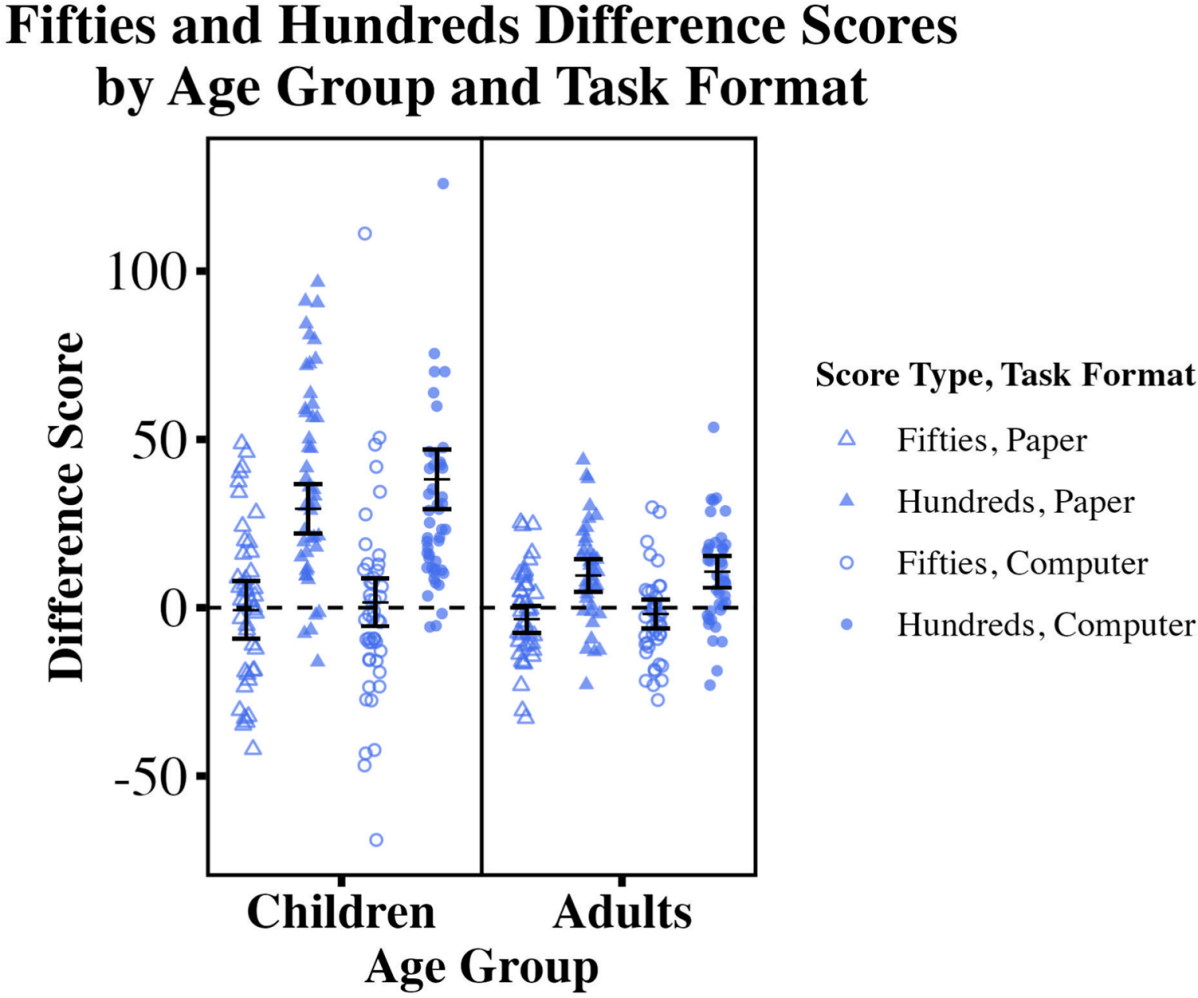
Children’s and adults’ fifties and hundreds difference scores for computer and paper tasks. Mean fifties and hundreds difference scores for each task format were generated from individual data from child and adult participants. Hundreds difference scores greater than zero indicate left digit effects. Individual and group mean difference scores and 95% confidence intervals are shown

We conducted an exploratory repeated-measures ANOVA on difference scores using within-participants factors pair type (fifties vs. hundreds) and task format (paper vs. computer), and between-participants factor age group (children vs. adults); see Fig. 6. We found a main effect of age (*F*(1, 81) = 28.65, *p* < .001, η2 = 0.075) and a main effect of pair type (*F*(1, 81) = 63.79, *p* < .001, η2 = 0.196): difference scores were larger in children than in adults, and hundreds scores (left digit effects) were larger than fifties scores. There was no main effect of task format (*F*(1,81) = 3.47, *p* = .066) when including both child and adult data in a single analysis. We found no interaction between task format and age (*F*(1,81) = 0.79, *p* = .378) and no interaction between task format and pair type (*F*(1,81) = 1.33, *p* = .253), but we did find an interaction between pair type and age (*F*(1,81) = 12.69, *p* < .001, η2 = 0.04): the difference between hundreds and fifties scores was larger for children. We found no overall three-way interaction (*F*(1,81) = 1.83, *p* = .180).

**Fig. 7:**
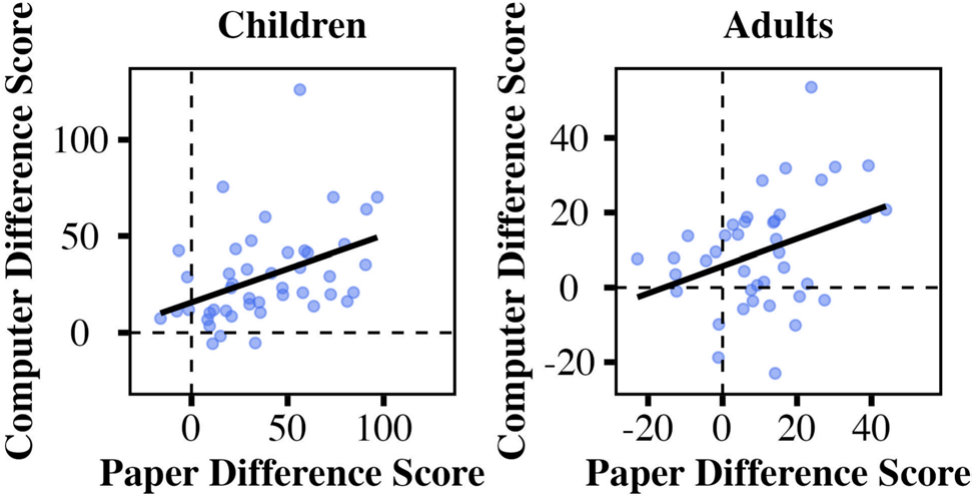
Correlation of left digit bias for paper and computer task formats. Both children’s and adults’ measures of left digit bias (mean hundreds difference scores) were correlated across task formats. Scales are different for children and adults. These graphs depict the same data shown in Fig. 5 in a different form

Exploratory analyses investigated the correlation of participants’ left digit effects (hundreds difference scores) across task formats (Fig. 7). Left digit biases for paper and computer tasks were positively correlated for both age groups (adults: *r* = .35, *p* = .026; children: *r* = .41, *p* = .005). Each age group had one participant with an unusually high hundreds difference score for the computer tasks; correlations remained significant with these two individuals excluded (adults: *r* = .32, *p* = .044; children: *r* = .45, *p* = .002).

## General discussion

The present studies aimed to extend our understanding of the generality, emergence, and development of left digit bias in number line estimation. Adults and children by roughly age 8 years consistently demonstrate left digit effects on both 0–100 and 0–1,000 number line tasks (Lai et al., 2018; Williams et al., 2022, 2023), sometimes with very large effect sizes. Under some conditions, large left digit effects have also been observed in 7-year-olds with a 0–1,000 task (Lai et al., 2018). Existing studies almost exclusively assessed left digit bias in numerical estimation with electronically presented versions of the number line task, with the exception of one experiment in which adults completed 0–100 number line tasks on paper (Savelkouls et al., 2020). One major goal of these studies was to ask whether a paper-based version would elicit left digit bias in the same way; this is important because paper-and-pencil number line tasks are common; number line placements are sensitive to slight task differences in multiple documented ways; and left digit bias can affect placement patterns enough to matter for potential interpretations of the data. Additionally, many published studies replicate left digit bias in adults’ number line placements but very few such child studies exist; the present work provides further exploration of left digit bias in children and its development. Finally, direct comparisons of left digit bias across paper and electronic task formats have not yet been made. In our first three studies, we asked whether children and adults display left digit effects when completing paper-and-pencil 0–100 and 0–1,000 number line estimation tasks; in a fourth study, we directly compared left digit bias for computer-based and paper-and-pencil task formats within participants for adults and children.

In Study 1, 8-year-olds’ placements were influenced by leftmost digits of target numerals. They placed numerals that were adjacent to a decade boundary (such as 49 and 51) farther apart on a paper 0–100 number line than would be predicted by their magnitudes alone, with the larger numeral placed to the right of the smaller. In contrast, 5- to 7-year-olds’ placements did not provide evidence of a left digit effect on the same task when measured in the same manner, consistent with prior work using computer-based tasks (Williams et al., 2022). Study 2 expanded the paper-based task to a larger numerical range of 0–1,000 and similarly found that children between the ages of 8 and 11 years show left digit bias, with large effect sizes, while 7-year-olds’ placements did not provide evidence of left digit bias at the group level. Study 3 demonstrated that adults also showed strong left digit effects on the same tasks, as evidenced by their placements of numerals on either side of a decade boundary (for 0–100) and on either side of a hundreds boundary (for 0–1,000). Together, these three studies offer further evidence that numerical estimation is heavily influenced by the identity of specific leftmost digits within multi-digit numbers in children and adults, in this case using paper-and pencil task formats, and provide additional support for previously reported patterns of emergence of the effect in children.

In Study 4, we directly compared paper and computer task formats within-participants in a formally preregistered pair of experiments. We used a 0–1,000 number line task with a new sample of children, in an age range that reliably exhibits left digit bias, and a new sample of adults. Large left digit effects were found in both age groups and in both task formats, consistent with the present Studies 2 and 3 and with other work. A key finding, and an unpredicted one, was that children’s left digit bias was larger for the paper task than for the computer task; adults’ bias showed no such difference. Therefore, at least for children aged 9–12 years on a 0–1,000 number line task, the task format does matter: on paper, left digit effects are enhanced relative to those observed in computer-based tasks.

In four groups of participants, we didn’t observe significant left digit bias: 5-, 6-, and 7-year-olds for the 0–100 task of Study 1 (contrasting with 8-year-olds) and 7-year-olds for the 0–1,000 task of Study 2 (contrasting with all other age groups, 8- through 11-year-olds). How should these results be interpreted? Could there be a left digit effect in some age groups that we cannot detect due to lack of power? For the youngest children, there is no support for this idea: the 5-year-olds’ mean difference scores are negative, incompatible with the direction of the effect. Similarly, the 6- and 7-year-olds have mean difference scores between 0 and 1 for the 0–100 task. To formally ask whether these data can support claims of a lack of effect, we used equivalence tests (Lakens, 2017; Lakens et al., 2018). These tests did not provide positive statistical evidence for the claim that there’s no left digit bias in these age groups. However, it is likely that these tests are underpowered in this case. In Study 2, it is more likely that the failure to observe a significant left digit effect in 7-year-olds in the 0–1,000 task could be due to a lack of power. Though left digit bias is usually a large effect, 7-year-olds’ performance is variable in this number range, and the mean hundreds difference score was greater than 20 (as one would expect, equivalence testing also can’t support a claim that there’s no left digit bias in this group). We also used equivalence tests to explore a different kind of lack of effect: in all studies, we calculated within-decade difference scores or fifties difference scores, comparing placements for numbers like 71 and 74, or 647 and 652. These types of pairs were never placed significantly too far apart with the larger numeral placed to the right, and mean scores often went in the opposite direction, supporting our claim that the left digit effect is fairly specific and that analogous effects are not seen in pairs that share left digits. We used equivalence tests to ask whether the data supported positive claims that these scores did not differ meaningfully from zero. No such claims could be made for the children in Studies 1 and 2, though power may be an issue in those cases. For the adults in Study 3, the data support the positive claim that fifties difference scores (but not within-decade difference scores) are not meaningfully different from zero. And for Study 4, which used larger samples, equivalence tests show that fifties difference scores are not meaningfully different from zero for both children and adults, and for both paper- and computer-based number line tasks. In general, these data do support the claim that there is no analogous effect to left digit bias occurring in other types of numeral pairs within a tens or hundreds range.

Evidence for left digit bias in children aged 7 or 8 years has been strong in some studies, and mixed or absent in others. In some cases, the absence of a significant group-level effect may be most likely due to power (a potential effect, but noisy data; see the 7-year-olds of the current Study 2). We think the best description of the overall pattern of results across studies, at least for English speakers in the USA, is that there is essentially no left digit bias in number line placements in 5- and 6-year-olds; that left digit bias is large and robust in adults and older children, observed reliably across task contexts, formats, and numerical ranges by the age of 9 years; and that it emerges roughly around age 7 or 8 years. Further studies will seek specific explanations of the emergence of left digit bias in children that cannot be addressed with current data. Of particular interest will be the identification of the elements of numerical understanding that lead to left digit bias in estimation. Some level of multidigit numeral facility and place value understanding must be a prerequisite, but are there certain operationalizations of place value understanding that best predict left digit bias? It is notable that in the present Study 1, the 5- and 6-year-old children who showed no trace of left digit bias on the 0–100 task did, nevertheless, did make distinctions between numbers within a tens range (e.g., placing numbers like 22 and 28 differently, on average). Five-year-olds, like those tested here, can generally perform the 0–100 task at least well enough to produce placements correlated with the target numerals, and they are on average sensitive to within-decade distinctions. At least this level of facility with two-digit numbers therefore appears to precede left digit bias, as far as we can tell from these cross-sectional group data. Children around age 5 and 6 are regularly given number line tasks in the 0–100 range, and they can carry them out reasonably well, but they do not yet exhibit any evidence of left digit bias; this remains to be explained by future studies. Investigation of potential links between left digit effects and children’s mathematical and verbal skills will also be valuable in future work (cf. Williams et al., 2020).

A particular developmental pattern in children’s left digit bias was reported in previously published work, but exploratory analyses of the present data found no evidence of this pattern. Experiments examining left digit effects in 0–1,000 number line estimation (Lai et al., 2018) found that the youngest children made number line placements based solely on hundreds digits, performing the task with reasonable success but without evidence of differentiating numerals with the same hundreds digits and very different magnitudes, like 701 and 798. Thus, not only did these children show left digit effects, but their placements were determined *solely* by leftmost digits. Slightly older children and adults also showed large left digit effects on the same task, but children aged 9 years and up successfully differentiated three-digit numbers with the same leftmost digit. However, a parallel developmental pattern was not observed in 0–100 tasks for a younger age range (Williams et al., 2022). In the present work, we also found no evidence of such a pattern in children in Study 1: 5- through 8-year-olds all successfully distinguished numbers with same left digits and different magnitudes, and only the 8-year-olds showed evidence of left digit bias. Nor did we find a parallel pattern to that of Lai et al. (2018) in the 0–1,000 paper-based number line task of the current Study 2. In Study 2, all age groups (ages 7–11 years) successfully differentiated numerals with the same hundreds digit but very different magnitudes, like 701 and 798, but the youngest children tested (7-year-olds) did not show evidence of a left-digit effect while older children did. The present study was not designed to explore this issue, but these data clearly show there is no reliably observed pattern in which children’s three-digit number line placements are initially based only on leftmost digits.

This work contributes to a growing collection of experiments exploring left digit bias in various forms of number line estimation across a range of ages and contexts. The left digit effect occurs in both standard, non-speeded number line estimation (e.g., the present studies; Lai et al., 2018, Experiment 2; Savelkouls et al., 2020, Study 2; Williams et al., 2022) and less typical speeded versions (Lai et al., 2018, Experiment 1). It occurs whether participants hear target numerals spoken out loud or not (Lai et al., 2018). It exists for both two-digit numbers in 0–100 tasks (e.g., the present work; Patalano et al. 2023, Savelkouls et al., 2020, Study 2; Williams et al., 2022) and three-digit numbers in 0–1,000 tasks (e.g., the present work; Lai et al., 2018; Williams et al, 2023). It is consistently observed by age 8 years in larger numerical ranges though not necessarily in smaller ones (the present studies; Lai et al., 2018, Williams et al., 2022) but appears as early as age 7 years in some contexts (Lai et al., 2018, Experiment 2). It remains present, with large effect sizes, in adults (the present work; Patalano et al., 2023; Savelkouls et al., 2020, Study 2; Williams et al., 2022, Study 2; Williams et al, 2023), even for non-canonical forms of the task with reverse-oriented 1,000-0 number lines (Williams et al., 2023). And although feedback can reduce other types of error in number line placements (e.g., Barth et al., 2016; Eyler et al., 2018; Opfer & Siegler, 2007), left digit bias in particular is highly resistant to interventions that attempt to reduce it, at least in adults (e.g., Gwiazda et al., 2024; Kayton et al., 2022; Williams et al., 2023). The sole exception to this general pattern of findings of robust left digit bias, to our knowledge, is that a left digit effect did not appear for 0–100 number line placements by Dutch-English bilingual adults when tested on paper in either language. This is perhaps because of the inverted place-value structure of tens and units in Dutch (e.g., “41” is “eenenveertig” – one and forty), suggesting that language structure could play an important role in the left digit effect (Savelkouls et al., 2020); however, further experiments, including studies in children, are needed to test this hypothesis more fully.

The strong left digit bias that is pervasive in number line estimation has implications for research employing this family of tasks, whether investigating its use as a pedagogical tool or as a probe of cognitive processes related to number and their development. To our knowledge, in prior work, left digit bias has generally not been accounted for when designing number line studies or when interpreting the results. Design, analysis, and interpretation usually focus on the overall numerical magnitudes of target numerals rather than on their constituent digits, in the sense that numbers like 799 and 802 in a 0–1,000 range, for example (or 79 and 80 in a 0–100 range) are often considered equivalent. Williams and colleagues (2022) highlighted two ways that these assumptions of equivalence could cause interpretation problems if left digit biases are present. One potential issue comes from the use of “contour analyses” as an assessment of the accuracy and variability of estimates near selected locations of the number line, like the midpoint and endpoints. With this type of approach, responses for target numerals like 48 and 52 on a 0–100 task, or 489 and 526 on a 0–1,000 task, may be averaged (e.g., Ashcraft & Moore, 2012; Jung et al., 2020; Peeters et al., 2016, Peeters, Verschaffel et al. 2017b). Averaging responses for target numerals with similar magnitudes and different left digits could obviously yield misleading results if done without recognition of the potential contributions of left digit bias. A related issue is that researchers often employ number line tasks in child studies that use a small number of target numerals. Left digit bias is likely to mean that the particular targets presented to a child could have a large but potentially underappreciated effect on their overall performance patterns.

Reliable findings of strong left digit effects across many studies, age groups, formats, and contexts show that assumptions of equivalence for numbers with similar magnitudes but different left digits are mistaken: they are not equivalent. In fact, placements are dramatically different for these numbers, with numbers below the relevant left digit boundary showing large leftward displacements (e.g., Kayton et al., 2022; Lai et al., 2018). Recently, Patalano and colleagues (2023) directly tested the idea that left digit bias is a result of leftward compression by collecting adults’ 0–100 number line placements for every value between 0 and 100. Analyses and modeling of placement data were consistent with a systematic pattern of compression such that placements within each tens range were shifted toward the left of the number line (e.g., placements of 21–29 compressed towards placements of 20), along with a general leftward compression across the entire number line. Parallel investigations will ask whether there is a similar pattern of leftward compression in children’s estimates and may help to further elucidate developmental sources of this bias as well as contributing to our understanding of underlying cognitive processes.

Overall, the present studies complement past findings, provide a needed replication of left digit bias in children’s estimation, further support and explore the broad developmental trajectory observed previously, and contribute two main novel results: the left digit effect occurs in both children and adults when the number line task is presented via paper and pencil, and paper-based tasks elicit a larger left digit bias in children. Number line estimation continues to be a widely used task in multiple subfields of psychological science. Left digit effects should be taken into account in the design and analysis of number line estimation tasks, and in the interpretation of the resulting data.

## Data Availability

Data and materials for the experiments reported here are not available. One of the four experiments was formally preregistered (Study 4).

## Footnotes

1 This method differs slightly from that of some other studies. Lai and colleagues did not subtract the absolute difference between paired numerals because they assumed that on a 0–1,000 number line, appropriate placements for two numerals that are only 2–4 units apart are presumably indistinguishable on a 24.5 cm number line with a 1,000-unit range (Lai et al., 2018). Results in the current study are the same using both methods.

2 Our preregistration specifies calculating PAE using a different method, but we report results using the same method as Studies 1–3.
